# The effect of systemic versus local transcutaneous laser therapy on tension-type cephalea and orofacial pain in post-COVID-19 patients: A pragmatic randomized clinical trial

**DOI:** 10.1097/MD.0000000000031218

**Published:** 2022-11-18

**Authors:** Mayra Costanti Vilela Campos, Silvana Simoes Velloso Schuler, Pamella de Barros Motta, Adriana Cátia Mazzoni, Francine Cristina da Silva, Manoela Domingues Martins, Kristianne Porta Santos Fernandes, Raquel Agnelli Mesquita-Ferrari, Anna Carolina Ratto Tempestini Horliana, Sandra Kalil Bussadori, Lara Jansiski Motta

**Affiliations:** a BioPhotonics Applied to Health Sciences Department, UNINOVE, São Paulo, Brazil; b Federal University of Bahia Multidisciplinary Institute of Health, Vitória da Conquista, Bahia, Brazil; c Oral Pathology and Oral Medicine, School of Dentistry, Federal University of Rio Grande do Sul, Porto Alegre, Brazil.

**Keywords:** laser therapycephalea, low power laser, orofacial pain, photobiomodulation

## Abstract

Orofacial pain and tensional cephalea were symptoms commonly reported in COVID-19 patients, even after recovery, and were considered chronic pain in these cases. The aim of this research is to evaluate the effect of the application of photobiomodulation with red and infrared lasers applied locally and systemically.

**Methods and analysis:**

For this purpose, individuals who have been diagnosed with COVID-19 and have had a tension headache and/or orofacial pain for more than 3 months will be selected by convenience. The participants will be divided into two different groups: G1-photobiomodulation with red and infrared laser with local application on the pain points (808 nm and 660 nm, 100 mW, 6 J per point) and G2-photobiomodulation with red laser with transcutaneous application on the radial artery (660 nm, 100 mW, 30 minutes). All participants will be treated for a period of 4 weeks, with 8 application sessions. The effects will be measured by means of blood lactate level, Brief Pain Inventory, Visual Analog Scale (VAS), and Cephalea Impact Test. The data will be collected weekly before and after the treatment, and the following tests will be applied: Analysis of variance (ANOVA), Tukey paired *t* test, Kruskal–Wallis, or Wilcoxon, according to data distribution. α = 0.05 will be considered as the level of statistical significance.

**Ethics and dissemination:**

This study was approved by the Research Projects Committee of the Nove de Julho University (approval number 4.673.963). Results will be disseminated through peer-reviewed journals and events for the scientific and clinical community, and the general public. It is registered in the ClinicalTrials.gov database with the number NCT05430776.

**Strengths and limitations of this study**

This study is an assessment for treatment for health impacts of the pandemic. This is a pragmatic study. Lasertherapy can be a non- invasive proposal for the treatment of pain resulting from COVID-19. There is not enough information in the literature on the relationship between laser therapy and post-covid-19 pain-related sequelae.

## 1. Introduction

Orofacial pain is defined as pain associated with tissues (skin, blood vessels, bones, teeth, glands, or muscles) of the oral cavity and face. Often, this pain can be referred to the head and/or neck region or even associated with cervicalgia, primary headaches, and rheumatic diseases.^[1,2]^

Cephalea refers to any referred pain in the cephalic segment and is a very common manifestation. During life, the prevalence of cephalea is higher than 90%, representing the third most common occurrence (10.3%) in neurology outpatient clinics. Cephalea is considered a public health problem, as they are debilitating disorders that sometimes make routine actions impossible, which causes a great personal and social impact.^[3,4]^

According to the International Classification of Cephalea, there are five predominant types of primary Cephalea, the most common being tension-type cephalea (TTH).^[3]^ This kind of pain is described as a pressure with mild to moderate intensity, bilateral.^[5]^

TTH is the most prevalent type worldwide, and the stiffness in the myofascial tissue in the cervical and cranial region strongly connects with the frequency and intensity of the discomfort. Genetic, emotional, and peripheral factors, such as nociception and central factors (central sensitization), are noted as etiological for orofacial pain.^[6]^

In addition to tension headache, temporomandibular disorders (TMD) are related to orofacial pain with signs and symptoms that include joint noises, limitations in range of motion or deviations during mandibular function, and temporomandibular joint pain.^[7,8]^

In 2020, the world was surprised by the pandemic of COVID-19, a disease caused by the coronavirus (SARS-CoV-2). The disease affected millions of people, causing many deaths. The clinical course of this new infection is characterized by respiratory symptoms and complications, however, cephalea is one of the signs frequently reported by COVID-19 patients.^[9]^

The cephalea attributed to the current viral infection is known as migraine or TTH, according to the 3rd edition of the International Classification of Headaches.^[10]^

It is estimated that with the pandemic COVID-19 there has been a 5-fold increase in the incidence of cephalea, and the most frequent pattern is bilateral. Other conditions were also evaluated the duration, frequency, course of the pain, and the relationship with a possible post-COVID-19 syndrome, as the disease has potential medium and long-term consequences.^[11]^

The worsening of the psycho-emotional state during the pandemic contributed not only to the increased incidence of cephalea but also to TMD-related pain.^[12,13]^

The importance of psychosocial factors in the development and maintenance of TMD is well established, and these factors have become more threatening to orofacial pain during the pandemic.^[14]^ However, in addition to psychosocial factors, the involvement of facial sensory innervation such as olfaction and taste. Although independent structures, there is an interrelationship between the olfactory nerve and the trigeminal nerve, contributing to the development and prolongation of orofacial pain.^[15]^

The recommended treatment for orofacial pain, as well as for cephalea, in general, aims at reducing the pain symptom and recovering the function. Pain control is often accomplished with the prescription of analgesics, anti-inflammatories, and other types of auxiliary drugs. However, in post-COVID-19 patients, pharmacological treatment may result in drug interactions resulting from the treatment for the viral infection. Considering this, the non-pharmacological auxiliary options that have the potential to contribute to pain relief can be considered.

One of the auxiliary treatment options for analgesia in TTH and TMD orofacial pain is photobiomodulation, that is, the use of a low-intensity laser or even light-emitting diode.

Low-intensity laser therapy (LILT) is being used frequently as a therapeutic procedure in a variety of medical situations, such as edema and inflammation control, chronic joint disorders, pain, and wound healing, among others.^[16–20]^

Chronic cephalea and cephalea associated with TMD have benefited from photobiomodulation for pain control. Therapeutic effects of LILT in TMD and headaches include an inflammatory modulator and analgesic effects.^[21–24]^ LILTs have demonstrated the ability to aid in the symptomatic treatment of pain, providing a considerable degree of comfort to patients right after their application. One of the main advantages of LILT is that it is a noninvasive, low-cost pain relief therapy. The use of laser therapy in patients with facial pain showed pain relief within minutes after its application, providing a significant well-being state. It is an auxiliary treatment for pain relief due to the analgesic action of the laser that allows the patient to resume their activities, providing more comfort and a better quality of life.^[21,22,24–26]^

Considering the auxiliary potential effect of photobiomodulation in controlling persistent TTH and TMD-related pain in patients who have been diagnosed with COVID-19, and are recovered from the viral infection, we intend to conduct a clinical trial comparing two modalities of therapeutic laser application: local application and transcutaneous application in the radial artery.

One of the main advantages of auxiliary techniques in pain control is the decrease of the use of drugs for analgesia, avoiding side effects and tolerance caused by them, and promoting an improvement in the individual’s quality of life.

## 2. Method

This is a pragmatic, randomized, parallel, single-blind clinical trial. The design follows the international recommendations for randomized clinical trials that are displayed in the SPIRIT Statement (Standard Protocol Items: Recommendations for Interventional Trials).

The research follows the regulatory norms established for human subjects research by the Research Ethics Committee of the Nove de Julho University with approval under number 4.673.963 that will periodically monitor the conduct of the study through semiannual reports. This is a low-risk study and symptom relief assessment does not require DMC. The participants will sign the free consent form after clarification, according to resolution 466/12 of the National Health Council. The recruitment procedures, eligibility assessment, and treatment will be carried out at UNINOVE’s Integrated Health Outpatient Clinics.

The selected subject will be individuals from the ages of 18 to 65 years, of both sexes, who have been diagnosed with COVID-19, confirmed by RT-PCR for SARS-CoV-2 already recovered at least 30 days from the infection, and present with orofacial pain will be selected.

Since this is a new scenario in health care and the information on the participants’ conditions is very recent, we chose a convenience sampling design. Considering a prevalence of persistent orofacial pain in infected participants of 11%,^[11]^ with a margin of error of 5%, reliability of 95%, and power of 80%, we intend to recruit, at least, 40 participants.

Participants will be invited to participate in the selection criteria that will follow the routine anamnesis and diagnosis of the International Classification of Headache and orofacial pain questionnaire.

### 2.1. Inclusion criteria

- Individuals of both sexes, between the ages of 19 and 65, complaining of persistent orofacial pain or functional-type cephalea, for more than 3 months.
- Individuals diagnosed with COVID-19, confirmed by RT-PCR for SARS-CoV-2 already recovered, at least 30 days after infection.

### 2.2. Exclusion criteria

- Individuals with diagnoses of neuropathies and headaches other than tension-type headache
- Physical or intellectual incapacity to answer the survey questionnaires; illiterate; diabetics, pacemakers, and pregnant women.
- Individuals who report photosensitivity to laser.

### 2.3. Discontinuation criteria

Participants who report any discomfort during the protocols, who present sensitivity to the laser application, or who do not attend more than two appointments in a row will be discontinued and the data will be computed for further analysis.

### 2.4. Study variables

Sociodemographic information will be collected, such as age, self-reported skin color, education, occupation, weight, height, and practice of physical activity. Besides the sociodemographic information, clinical information will be collected, such as the history of systemic diseases and personal and family history of health problems, treatments performed previously and at the time of the survey, and medications used.

### 2.5. Pain scale

The Brief Pain Inventory (BPI) is a multidimensional instrument that uses a 0 to 10 scale to grade the following items: intensity, interference of pain in the participant’s ability to walk, daily activities, work, social activities, mood, and sleep. The pain rated by the participant is that experienced at the time of the questionnaire and the most intense, least intense, and average pain of the last 24 hours. The Brief Pain Inventory scale will be applied weekly.

Visual Analog Scale for pain: one-dimensional instrument for pain intensity assessment. It is a line with the ends numbered from 0 to 10. At one end of the line is marked “no pain” and at the other end “worst pain imaginable.” The participant is then asked to evaluate and mark on the line the pain present at that moment. The Visual Analog Scale will be applied at the beginning and the end of each session.

Headache Impact Test (HIT-6) – To evaluate the impact of the protocols used in the treatment of headache on the participants’ quality of life, the Headache Impact Test known as HIT-6 applied at the four moments of the participant’s evaluation, according to the study protocol. It is an easy-to-apply test, reliable, and validated for the Brazilian reality.

The test consists of six questions, covering the domains of pain, ability to perform usual activities, social functioning, energy/fatigue, cognition, and emotional stress. Scores are calculated by assigning a value of 6 points when the answer is “never,” 8 points for “rarely,” points for “sometimes,” points for “very often” and 3 points when the answer is “always.” The score ranges from 36 to 78. If the score is <49 points, they suggest that the headache has little or no impact on the participant’s quality of life. If the score is 50 to 55 points, it means that there may be an impact on the daily activities, but the individual is still able to perform the activities normally. Scores between 56 to 59 points constitute substantial impact, that is, there is difficulty in performing simple daily activities due to the pain. Scores >60 points imply a very intense impact on quality of life, leading to the inability to perform daily activities. The HIT-6 test will be applied at the beginning and the end of the treatment.

### 2.6. Blood lactate level

For blood collection, a disposable and sterile lancet will be used. A small prick will be made on the right earlobe, and the collected blood (25 µL) will be deposited on a reactive tape to be analyzed in a portable lactometer (Detect TD-4261 Lactate Analyzer, EcoDiagnóstica, MG/Brazil, vendas@ecodiagnostica.com.br). The lactate level will be collected once a week, before and after the application of the protocol. The lactate level is related to muscle activity and may help in the understanding of the effect of photobiomodulation in this research.

The need for medication changes or the introduction of new drugs other than the usual ones will also be collected by means of a medication recall.

### 2.7. Research conduct and protocol

The treatment will be carried out in 8 weeks (2 sessions per week) with an average duration of 60 minutes per session. At the end of the 8-week treatment, a new evaluation will be performed after 1 month of follow-up.

After selection, the participants will be randomized into two groups: (G1) photobiomodulation with red and infrared laser with local application on pain points and (G2) photobiomodulation with red laser with transcutaneous application in the radial artery.

The draw for allocation to the groups will be carried out by means of generating number sequences on the electronic platform www.randon.org. The sequence will be kept in a sealed opaque envelope. Only one researcher will have access to the sequencing.

**Figure 1.**
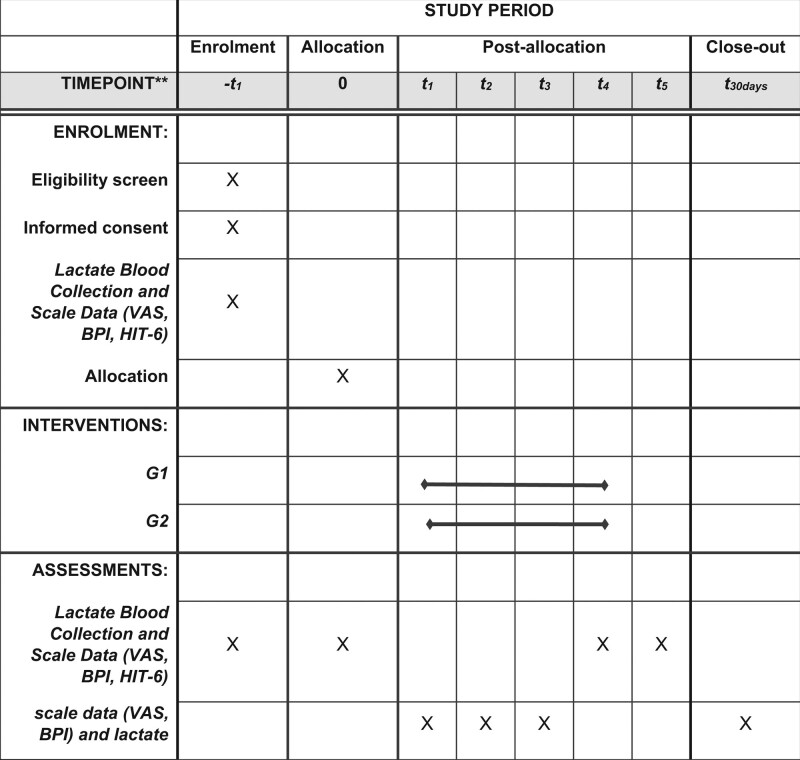
Schedule of enrollment, interventions, and assessments. This clinical trial will follow the consort (Consolidated Standards of Reporting Trials) recommendations. Figure 1 presents the flow chart of the research. BPI = Brief Pain Inventory, HIT-6 = Headache Impact Test, VAS = Visual Analog Scale.

After allocation, the participants will answer the evaluation scales and the blood will be collected for analysis of the initial lactate level (Fig. 1).

The participants will be positioned on a clinical chair or stretcher for the application of photobiomodulation at the Universidade Nove de Julho dental clinic. The interventions will be performed by trained professionals.

G1 will receive the application of photobiomodulation with the device The Ecco Reability (Eccofibras, São Paulo, Brazil) containing its infrared wavelength of 808 ± 10 nm and red 660 ± 10 nm concomitant and power 100 mW, properly calibrated and energy of 6 J per point (60 seconds) in 8 points, 2 sessions per week in 8 points on the face and neck. The points will be those referred to by the participant as pain or trigger points located during the clinical examination. G2 will receive the application of photobiomodulation with the device The Ecco Reability (Eccofibras) containing its red wavelength 660 ± 10 nm concomitant and power of 100 mW, with an application time of 30 minutes (Table 1).

**Table 1:** Dosimetric parameters of photobiomodulation application.

Parameters	Red laser (Local)	Red laser (Local)	Red laser (systemic transcutaneous)
Wavelength (nm)	808	660	660
Operation mode	Continuous	Continuous	Continuous
Power (mW)	100	100	100
Opening diameter (cm)	0.354 (beam diameter with spacer)	0.354 (beam diameter with spacer)	
Beam area (cm^2^)	0.0984 (with spacer)	0.0984 (with spacer)	
Exposure time (s)	60 per point	60 per point	1800
Fluency (J/cm^2^)	61	61	–
Energy (J)	6 per point	6 per point	180J
Number of irradiated points	8	8	Systemic
Application technique	Contact	Contact	Contact
Session number	8	8	8
Treatment frequency	2 times a week	2 times a week	2 times a week
Total energy radiated (J)	384 J	384 J	1440 J

The device will be positioned with the spot located on the radial artery of the participant’s preferred arm (right or left) and attached to the wrist with a specific wristband. There will be 8 transcutaneous applications, 2 sessions per week of 30 minutes each session.

Any changes made to the protocol will be communicated to the participants using the media of Universidade Nove de Julho and the academic community involved through attachments in the original project.

### 2.8. Statistical analysis

The data will be presented descriptively as to the sociodemographic variables and clinical characteristics of the sample. The data of the clinical variables will be analyzed by statistical software SPSS 23.0 for Win. After the normality test, Analysis of variance (ANOVA) followed by Tukey’s test will be applied when necessary and for the treatment results in the study periods, the paired *t* test will be used. If the hypothesis of normality is rejected, the Kruskal–Wallis test will be used followed by Student’s test, and for comparison in the different study periods, the Wilcoxon test will be used. We will take α = 0.05 as the level of statistical significance.

### 2.9. Analysis, data management, and dissemination

The data collected during the research will be stored and organized in the Harvard Dataverse repository (https://dataverse.harvard.edu). The metadata will be published on the site in the repository via the electronic address provided by the platform (DOI).

The data concerning the participants and the research outcomes will remain confidential. Only researchers will have access to this information. Upon completion of the research, the data will be published and disseminated in national and international scientific events and journals.

### 2.10. Patient and public envolviment

The development of this project relied on a multidisciplinary team from Universidade Nove de Julho. The results will be discussed with the study groups involved and will also be disseminated to the participants who requested them and will be published in the newsletters of Universidade Nove de Julho.

## 3. Discussion

Pandemic COVID-19 is shown to be a potential factor in the development, worsening, or persistence of TTH and/or orofacial pain.^[5,9,10,12,13]^

Explanatory studies evaluate “if” and “how” the intervention works when applied to participants (efficacy). However, the observed benefits of the interventions may be delimited to a very particular category of participants who met the strict criteria for inclusion or selective exclusion of these patients.^[27]^ When the study intends to evaluate effectiveness, the intervention, already with previously proven effectiveness, should be tested in “real world” conditions.^[26,27]^

This research with a pragmatic trial is justified to respect the features of multimodal treatment of orofacial pain and promote improvement in the quality of life of individuals with TTH and TMD since the literature has already demonstrated the effectiveness of photobiomodulation in explanatory trials.

The aim is to reproduce the conditions found in the clinical routine, since simplified procedures but complex elaboration, derive few or no changes in the clinician’s work routines. The control of variables in the analyses of the results will be done using statistical tests and subgroup evaluations. This pragmatic clinical trial should be seen as complementary to explanatory trials in producing evidence and aiding clinical decision-making.

Persistent symptoms after COVID-19 infection should be treated with care and concern for the patient’s well-being. The results of this study may support clinical choices and decision-making regarding noninvasive and non-pharmacological adjuvants for the control of tension-type headache and TMD-related orofacial pain. It is expected that the tested protocols will be evaluated for their effect on analgesia, compliance to treatment by the patient, and comfort in the application. The results will serve as input for the development of clinical protocols and therapeutic guidelines.

## Author contributions

**Conceptualization:** Mayra Costanti Vilela Campos, Kristianne Porta Santos Fernandes, Raquel Agnelli Mesquita Ferrari, Anna Carolina Ratto Tempestini Horliana, Sandra Kalil Bussadori, Lara Jansiski Motta.

**Designed the study and wrote the study protocol:** Mayra Costanti Vilela Campos, Silvana Simoes Velloso Schuler, Pamella de Barros Motta, Adriana Cátia Mazzoni, Kristianne Porta Santos Fernandes, Raquel Agnelli Mesquita Ferrari, Anna Carolina Ratto Tempestini Horliana, Sandra Kalil Bussadori, Lara Jansiski Motta.

**Funding acquisition:** Mayra Costanti Vilela Campos and Lara Jansiski Motta.

**Methodology:** Mayra Costanti Vilela Campos, Pamella de Barros Motta, Francine Cristina da Silva, Manoela Domingues Martins, Lara Jansiski Motta.

**Project administration:** Mayra Costanti Vilela Campos, Silvana Simoes Velloso Schuler, Adriana Cátia Mazzoni, Lara Jansiski Motta.

**Writing – review & editing:** Mayra Costanti Vilela Campos, Silvana Simoes Velloso Schuler, Pamella de Barros Motta, Adriana Cátia Mazzoni, Francine Cristina da Silva, Manoela Domingues Martins, Kristianne Porta Santos Fernandes, Raquel Agnelli Mesquita Ferrari, Anna Carolina Ratto Tempestini Horliana, Sandra Kalil Bussadori, Lara Jansiski Motta.
